# Use of PETRA-MRA to assess intracranial arterial stenosis: Comparison with TOF-MRA, CTA, and DSA

**DOI:** 10.3389/fneur.2022.1068132

**Published:** 2023-01-16

**Authors:** Junxia Niu, Yuncai Ran, Rui Chen, Feifei Zhang, Xiaowen Lei, Xiao Wang, Tengfei Li, Jinxia Zhu, Yong Zhang, Jingliang Cheng, Yan Zhang, Chengcheng Zhu

**Affiliations:** ^1^Department of Magnetic Resonance, The First Affiliated Hospital of Zhengzhou University, Zhengzhou, China; ^2^Department of Magnetic Resonance, Pingmei Shenma Medical Group General Hospital, Pingdingshan, China; ^3^Department of Magnetic Resonance, Xuchang Central Hospital, Xuchang, China; ^4^Department of Intervention, The First Affiliated Hospital of Zhengzhou University, Zhengzhou, China; ^5^MR Collaboration, Siemens Healthineers Ltd., Beijing, China; ^6^Department of Radiology, University of Washington, Seattle, WA, United States

**Keywords:** computed tomography angiography, 3D time-of-flight magnetic resonance angiography, digital subtraction angiography, intracranial arterial stenosis, pointwise encoding time reduction magnetic resonance angiography

## Abstract

**Background and purpose:**

Non-invasive and accurate assessment of intracranial arterial stenosis (ICAS) is important for the evaluation of intracranial atherosclerotic disease. This study aimed to evaluate the performance of 3D pointwise encoding time reduction magnetic resonance angiography (PETRA-MRA) and compare its performance with that of 3D time-of-flight (TOF) MRA and computed tomography angiography (CTA), using digital subtraction angiography (DSA) as the reference standard in measuring the degree of stenosis and lesion length.

**Materials and methods:**

This single-center, prospective study included a total of 52 patients (mean age 57 ± 11 years, 27 men, 25 women) with 90 intracranial arterial stenoses who underwent PETRA-MRA, TOF-MRA, CTA, and DSA within 1 month. The degree of stenosis and lesion length were measured independently by two radiologists on these four datasets. The degree of stenosis was classified according to DSA measurement. Severe stenosis was defined as a single lesion with >70% diameter stenosis. The smaller artery stenosis referred to the stenosis, which occurred at the anterior cerebral artery, middle cerebral artery, and posterior cerebral artery, except for the first segment of them. The continuous variables were compared using paired *t*-test or Wilcoxon signed rank test. The intraclass correlation coefficients (ICCs) were used to assess the agreement between MRAs/CTA and DSA as well as inter-reader variabilities. The ICC value >0.80 indicated excellent agreement. The agreement of data was assessed further by Bland–Altman analysis and Spearman's correlation coefficients. When the difference between MRAs/CTA and DSA was statistically significant in the degree of stenosis, the measurement of MRAs/CTA was larger than that of DSA, which referred to the overestimation of MRAs/CTA for the degree of stenosis.

**Results:**

The four imaging methods exhibited excellent inter-reader agreement [intraclass correlation coefficients (ICCs) > 0.80]. PETRA-MRA was more consistent with DSA than with TOF-MRA and CTA in measuring the degree of stenosis (ICC = 0.94 vs. 0.79 and 0.89) and lesion length (ICC = 0.99 vs. 0.97 and 0.73). PETRA-MRA obtained the highest specificity and positive predictive value (PPV) than TOF-MRA and CTA for detecting stenosis of >50% and stenosis of >75%. TOF-MRA and CTA overestimated considerably the degree of stenosis compared with DSA (63.0% ± 15.8% and 61.0% ± 18.6% vs. 54.0% ± 18.6%, *P* < 0.01, respectively), whereas PETRA-MRA did not overestimate (*P* = 0.13). The degree of stenosis acquired on PETRA-MRA was also more consistent with that on DSA than with that on TOF-MRA and CTA in severe stenosis (ICC = 0.78 vs. 0.30 and 0.57) and smaller artery stenosis (ICC = 0.95 vs. 0.70 and 0.80). In anterior artery circulation stenosis, PETRA-MRA also achieved a little bigger ICC than TOF-MRA and CTA in measuring the degree of stenosis (0.93 vs. 0.78 and 0.88). In posterior artery circulation stenosis, PETRA-MRA had a bigger ICC than TOF-MRA (0.94 vs. 0.71) and a comparable ICC to CTA (0.94 vs. 0.91) in measuring the degree of stenosis.

**Conclusion:**

PETRA-MRA is more accurate than TOF-MRA and CTA for the evaluation of intracranial stenosis and lesion length when using DSA as a reference standard. PETRA-MRA is a promising non-invasive tool for ICAS assessment.

## Introduction

Intracranial arterial stenosis (ICAS) is one of the leading causes of ischemic stroke and associated morbidity and mortality worldwide (1). The assessment of the degree of stenosis and lesion length in the intracranial artery is critical for patient management and treatment planning (2). Digital subtraction angiography (DSA) is regarded as the gold standard for measuring intracranial stenosis. However, DSA is an invasive modality and has the risk of ionizing radiation and the incidence of potential subsequent contrast-related complications (3). Therefore, non-invasive and accurate methods for the assessment of ICAS are gaining promise.

The current traditional non-invasive assessment methods of ICAS include computed tomography angiography (CTA), 3D time-of-flight magnetic resonance angiography (TOF-MRA), and contrast-enhanced MRA. Among them, CTA and 3D TOF-MRA are popular techniques that are routinely used in clinical practice. CTA has been regarded widely as the first choice owing to the less expensive, high spatial resolution, and fast imaging with good accuracy. 3D TOF-MRA is the most widely used technique for various intracranial vascular evaluations without radiation and contrast injection. However, CTA is restricted by contrast-related clinical complications such as nephropathy or exposure to radiation (4–6), and TOF-MRA has limited accuracy for measuring the degree of stenosis because of the flow-related dephasing artifacts (7). CTA and TOF-MRA commonly overestimate the degree of stenosis in severe stenosis. 3D pointwise encoding time reduction MRA (PETRA-MRA) is an emerging non-contrast-enhanced MRA method. In addition, as an ultrashort echo time sequence, PETRA-MRA is less sensitive to turbulent or slow flow artifacts and attenuates acoustic noise, resulting in good signal-to-noise ratio (SNR) of the flowing lumen and signal homogeneity (8–10). PETRA-MRA has been used in the evaluation of intracranial aneurysms (11, 12) but very few studies applied such a technique in the assessment of intracranial stenosis (13). No study has compared PETRA-MRA with TOF-MRA and CTA directly in the same cohort, using DSA as a reference standard. Therefore, this prospective study aimed to compare the accuracy and reproducibility of PETRA-MRA to TOF-MRA and CTA, using DSA as the reference standard in measuring the degree of stenosis and lesion length in ICAS.

## Materials and methods

### Patients

This single-center prospective study was approved by the local institutional review board, and informed consent was obtained from all participants. The patients who presented between October 2017 and September 2022 and underwent DSA for the suspected cerebrovascular disease were recruited. The inclusion criteria were as follows: (1) patients with stroke or transient ischemic attack attributing to ICAS, (2) patients aged older than 18 years, and (3) patients undergoing MRAs (including PETRA-MRA and TOF-MRA), CTA, and DSA, and the interval between the four imaging sessions being within 1 month. Patients were excluded if they had any of the following conditions: (1) intracranial hemorrhage or non-stenotic intracranial vasculopathy, (2) complete intracranial artery occlusion, (3) inadequate image quality, and (4) intracranial artery with a long-segment or too tortuous stenosis that could not be accurately measured the lesion length.

### Digital subtraction angiography

Digital subtraction angiography examinations were performed on a fixed digital angiographic system, FD 20 Artis (Phillips Healthcare, Best, The Netherlands). All patients underwent local anesthesia and femoral artery catheterization. 2D DSA acquisition protocol was performed with Omnipaque350 contrast injection (GE Healthcare, WI, USA) at a rate of 4 mL/s. The scan parameters were as follows: field of view (FOV) = 320 × 320 mm^2^; matrix = 1,024 × 1,024. Four-vessel angiography was performed in all patients. Standard anteroposterior, oblique, and lateral views were obtained for all interrogated arteries.

### MRI protocol

All patients underwent MRAs on a 3.0 T system (MAGNETOM Prisma, Siemens Healthcare, Erlangen, Germany) with a 64-channel head-neck coil.

The detailed parameters for TOF-MRA were as follows: repetition time (TR)/echo time (TE) = 20/3.69 ms; acquisition plane axial; FOV = 200 × 160 mm^2^; matrix = 320 × 256; slice thickness = 0.6 mm; voxel size = 0.6 × 0.6 × 0.69 mm^3^; number of slices = 176; and acquisition time = 3 min 29 s. The detailed parameters for PETRA-MRA were as follows: TR/TE = 3.32/0.07 ms; FOV = 300 × 300 mm^2^; matrix = 320 × 320; slice thickness = 0.9 mm; voxel size = 0.9 × 0.9 × 0.9 mm^3^; radial views number = 60,000; and number of slices = 320. A labeled scan (with a saturation band proximal to the imaging volume) and a control scan (with the saturation band above the head vertex) were acquired. The total acquisition time of PETRA-MRA was 9 min and 20 s. The PETRA-MRA images were subtracted from the two scans (control-labeled).

### CTA protocol

Computed tomography angiography head/neck scanning protocol was performed on the 128-multislice Siemens Somatom AS + scanner (Siemens Healthcare, Erlangen, Germany). Approximately 100 mL of Omnipaque350 contrast (GE Healthcare, WI, USA) was injected at a rate of 5 mL/s followed by 30 mL of normal saline flush injected at the same rate. Scanning was performed from the aortic arch through the vertex. The scanning parameters were as follows: quality reference = 250 mAs; tube voltage = 120 kV; pitch = 0.9; rotation time = 0.5; collimation = 128 × 0.6 mm^2^; slice thickness: 0.6 mm; FOV = 300 × 300 mm^2^; matrix = 512 × 512; and voxel size = 0.35 × 0.35 × 0.5 mm^3^.

### Image analysis

The maximum intensity projections (MIPs) of MRAs and CTA images were performed by a neuroradiologist (over 5 years of experience) at Siemens workstation. The MRAs and CTA datasets were anonymized and placed in random order. The MIP images of the MRAs and CTA were reviewed and evaluated by two radiologists (over 10 years of experience and 8 years of experience, respectively) using a 4-point scale (4 = excellent, 3 = good, 2 = poor, and 1 = not recognizable). The datasets with poor image quality (score ≤2) were excluded from the analysis. The degree of stenosis and lesion length on DSA and the MIPs of MRA and CTA were measured by the same two radiologists independently, blind to the patient's clinical information.

The diameter of stenosis (minimum lumen diameter) referred to the diameter of the residual lumen at the maximal narrowing site. The diameter of the normal segment was measured proximal to the site of maximal luminal narrowing. The lesion length was measured as stenosis length on MRAs, CTA, and DSA, which is the distance from the proximal to the distal of stenosis along with the virtual center of the artery (14).

The degree of stenosis was measured according to warfarin–aspirin symptomatic intracranial disease criteria (15) as follows:


$$\begin{array}{l} Stenosis\%=\left(1-\text{d}/\text{D}\right)\times \;100\% \end{array}$$

where *d* is the diameter at the maximal stenosis, and *D* is the diameter of the proximal normal segment.

Severe stenosis was defined as a single lesion with >70% diameter stenosis (16). The smaller artery stenosis referred to the stenosis, which occurred at the anterior cerebral artery, middle cerebral artery, and posterior cerebral artery except for the first segment of them.

### Statistical analysis

The normality of continuous variables was tested with Shapiro–Wilk's test. Continuous variables were expressed as the mean ± standard deviation (SD) or median (interquartile range) and compared by paired *t*-test or Wilcoxon signed rank test, respectively. The image quality scores were compared using the Kruskal–Wallis test.

The intraclass correlation coefficient (ICC) was used to assess the inter-reader variabilities and the measurement agreement between MRAs/CTA and DSA not only in all stenoses but also in different stenosis types, such as the severe stenosis, smaller artery stenosis, and anterior/posterior cerebral circulation stenosis. The ICC value of >0.80 indicated excellent agreement. The agreement of data satisfied normality was assessed further by Bland–Altman analysis. Bias was assessed as the mean of the paired differences, and the 95% limits of agreement (LOA) were defined as bias ± 1.96 × SD. Measurement error was quantified by the coefficient of variance (CV = SD of difference/mean × 100%). The performances of TOF-MRA, PETRA-MRA, and CTA in detecting stenosis of >50% and stenosis of >75% were summarized by the sensitivity, specificity, positive predictive value (PPV), and negative predictive value (NPV), with DSA as the reference standard. All statistical analyses were performed using SPSS 24.0 (IBM Corp., NY, USA) software or GraphPad Prism 8 (GraphPad Software, CA, USA). The statistical significance was set at *P* < 0.05, and *P*-values were two-sided.

## Results

### Patient demographics and imaging findings

**Figure 1:**
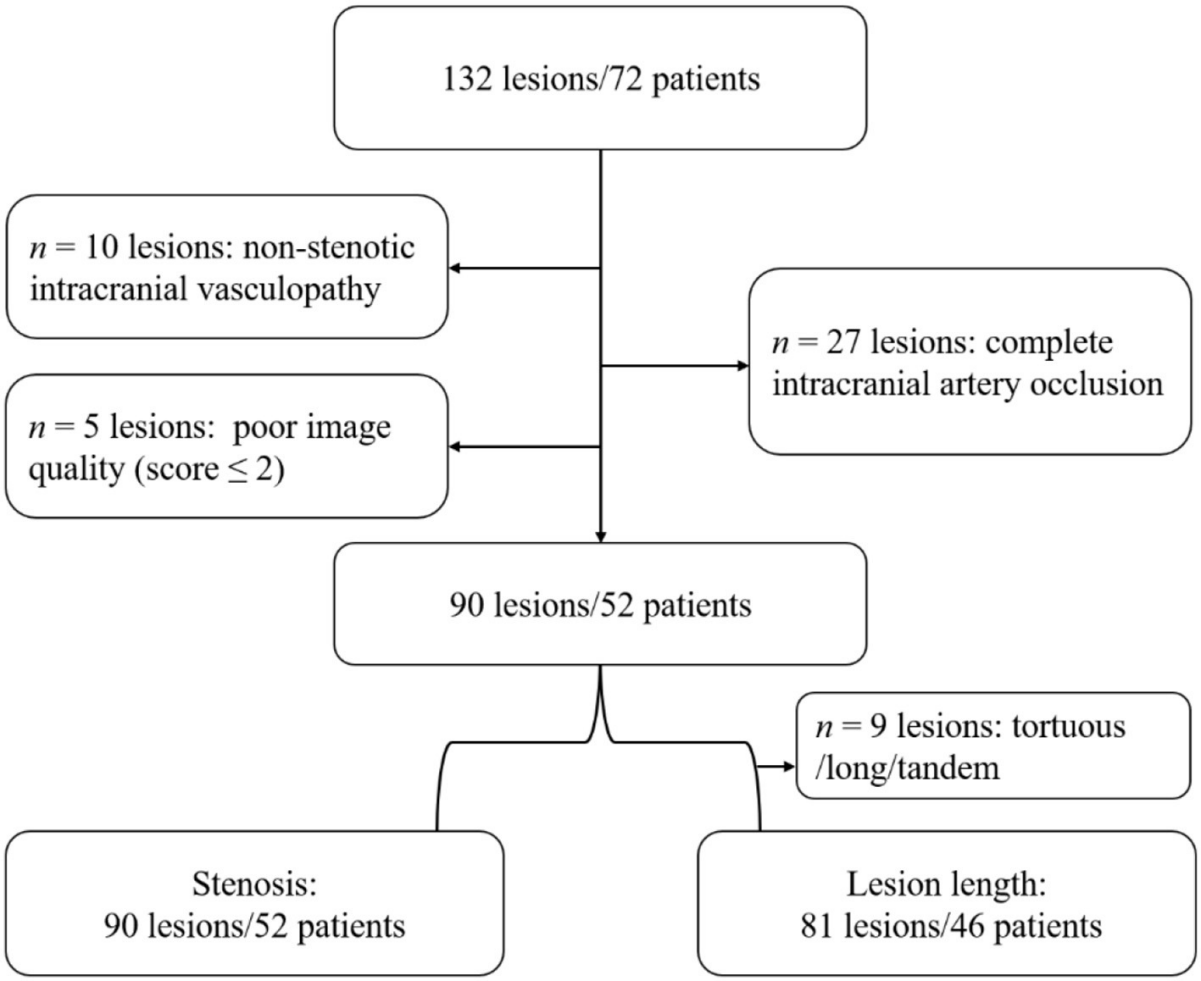
Flowchart of study population selection.

In an initial cohort of 132 lesions in 72 patients, 10 patients had non-stenotic intracranial vasculopathy, 27 lesions had complete occlusion, and five patients had poor image quality (score ≤2). Moreover, nine lesions were too long/torturous to accurately measure the lesion length. Finally, 90 lesions in 52 patients were included in the degree of stenosis analysis, and 81 lesions in 46 patients were included in the lesion length analysis (Figure 1).

The anterior circulation had 71 stenoses, including nine stenoses in the anterior cerebral artery (ACA), 55 in the middle cerebral artery (MCA), and seven in the intracranial internal carotid artery (ICA). The posterior circulation had 19 stenoses, including six stenoses in the basilar artery (BA), five in the vertebral artery (VA), and eight in the posterior cerebral artery (PCA). The severe stenosis (>70%) has 24 stenoses (four stenoses in ACA, 17 in MCA, two in ICA, and one in PCA). There were 14 stenoses in smaller arteries (two stenoses in the A2 segment of ACA and 12 in the M2 segment of MCA). The patients had no history of balloon dilatation or stent implantation. The time interval was 4 ± 3 days between DSA and MRAs and 44 ± 50 days between symptom onset and MRAs. The time interval was 9 ± 4 days between DSA and CTA and 40 ± 50 days between symptom onset and CTA. Image scores showed excellent image quality for PETRA-MRA (3.97±0.18), TOF-MRA (3.94±0.23), and CTA (3.97±0.18), with no significant differences among them (*P* = 0.69).

**Figure 2:**
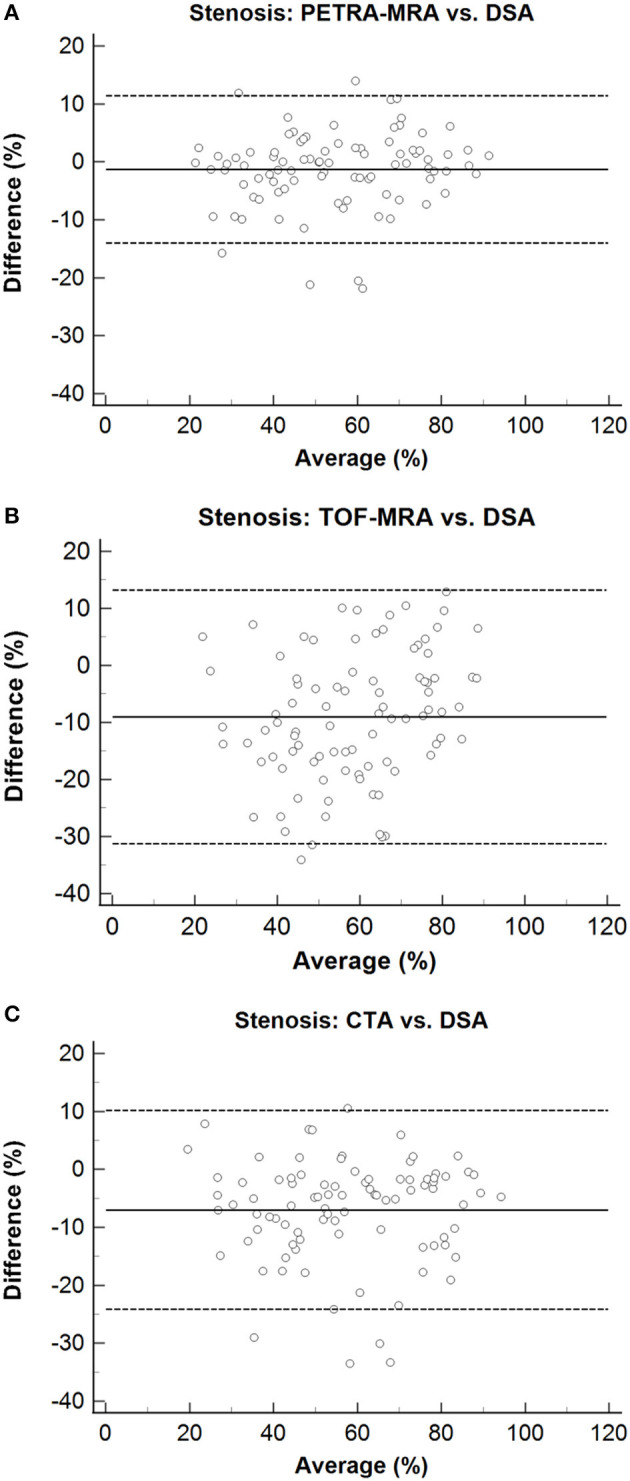
Bland–Altman plots for stenosis of PETRA-MRA, TOF-MRA, and CTA, compared with the DSA as the reference standard. The solid lines represent the mean difference, and the dashed lines indicate the 95% confidence interval. The measurements of the degree of stenosis on PETRA-MRA indicated the smallest variance with DSA and shortest LOA compared with those on TOF-MRA and CTA [CV: 11.8% vs. 19.4% and 15.2%; LOV: (−14.0, 11.4) vs. (−31.3, 13.2) and (−24.1.10.2)]. **(A)** Stenosis: PETRA-MRA vs. DSA, **(B)** Stenosis: TOF-MRA vs. DSA, **(C)** Stenosis: CTA vs. DSA.

**Figure 3:**
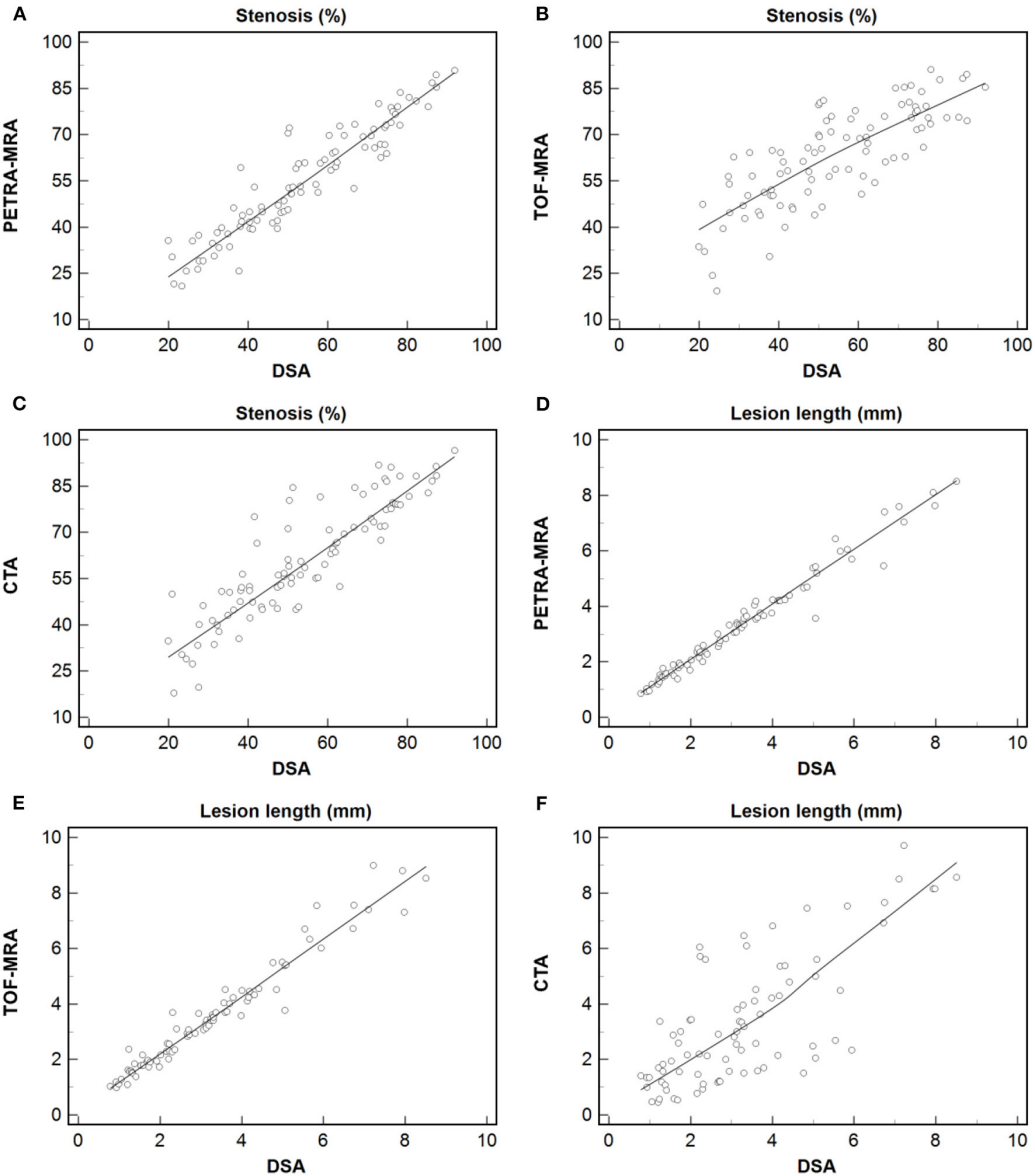
Measurement of the degree of stenosis and lesion length using PETRA-MRA, TOF-MRA, or CTA vs. those using DSA. Dashed line = regression line. The greatest correlation was observed in measuring the degree of stenosis and lesion length between PETRA-MRA and DSA than between TOF-MRA and DSA, and between CTA and DSA [r (stenosis): 0.94 vs. 0.80 and 0.89; r (lesion length): 0.99 vs. 0.98 and 0.70]. **(A–C)** Stenosis: Spearman's correlation analysis of PETRA-MRA, TOF-MRA, and CTA with DSA respectively. **(D–F)** Lesion length: Spearman's correlation analysis of PETRA-MRA, TOF-MRA, and CTA with DSA respectively.

The agreement between PETRA-MRA, TOF-MRA, CTA, and DSA on the measurements of the degree of stenosis and lesion length is summarized in Table 1. PETRA-MRA achieved a little bigger ICC than TOF-MRA and CTA in measuring the degree of stenosis (0.94 vs. 0.79 and 0.89), and PETRA-MRA achieved the comparable ICC to TOF-MRA (0.99 vs. 0.97) and the bigger ICC than CTA in measuring lesion length (0.99 vs. 0.73). The measurements of the degree of stenosis on PETRA-MRA indicated the smallest variance with DSA (CV: PETRA-MRA = 11.8%, TOF-MRA = 19.4%, CTA = 15.2%) and narrowest LOA compared with those on TOF-MRA and CTA [LOV: PETRA-MRA = (−14.0, 11.4), TOF-MRA = (−31.3, 13.2), CTA = (−24.1, 10.2)] (Figure 2). The correlations of the measurements of the degree of stenosis and lesion length between PETRA-MRA and DSA were greatest than those between TOF-MRA and DSA and between CTA and DSA [*r* (stenosis): PETRA-MRA = 0.94, TOF-MRA = 0.80, CTA = 0.89; *r* (lesion length): PETRA-MRA = 0.99, TOF-MRA = 0.98, and CTA = 0.70] (Figure 3).

**Table 1:** Comparison of MRAs/CTA and DSA in measuring the degree of stenosis and lesion length.

	**DSA**	**PETRA-MRA**	**TOF-MRA**	**CTA**
**Stenosis (100%)**				
Mean ± SD	54.0 ± 18.6	55.3 ± 17.7	63.0 ± 15.8	61.0 ± 18.6
CV (%)	Reference	11.8	19.4	15.2
*P*	Reference	0.13	< 0.01	< 0.01
Bias	Reference	−1.3	−9.0	−7.0
LOA	Reference	(−14.0, 11.4)	(−31.3, 13.2)	(−24.1, 10.2)
*r*	Reference	0.94	0.80	0.89
*ICC*	Reference	0.94	0.79	0.89
**Lesion length (mm)**				
Median	3.13 (2.12, 4.50)^&^	3.31 (2.06, 4.27)^&^	3.41 (2.25, 4.50)^&^	3.05 (1.96, 5.34)^&^
*P*	Reference	0.06	0.17	0.78
*r*	Reference	0.99	0.98	0.70
*ICC*	Reference	0.99	0.97	0.73

& Interquartile range. CTA, computed tomography angiography; CV, coefficient of variation; DSA, digital subtraction angiography; ICC, intraclass coefficient; LOA, limit of the agreement; MRA, magnetic resonance angiography; PETRA, pointwise encoding time reduction; SD, standard deviation; TOF, time of flight.

**Figure 4:**
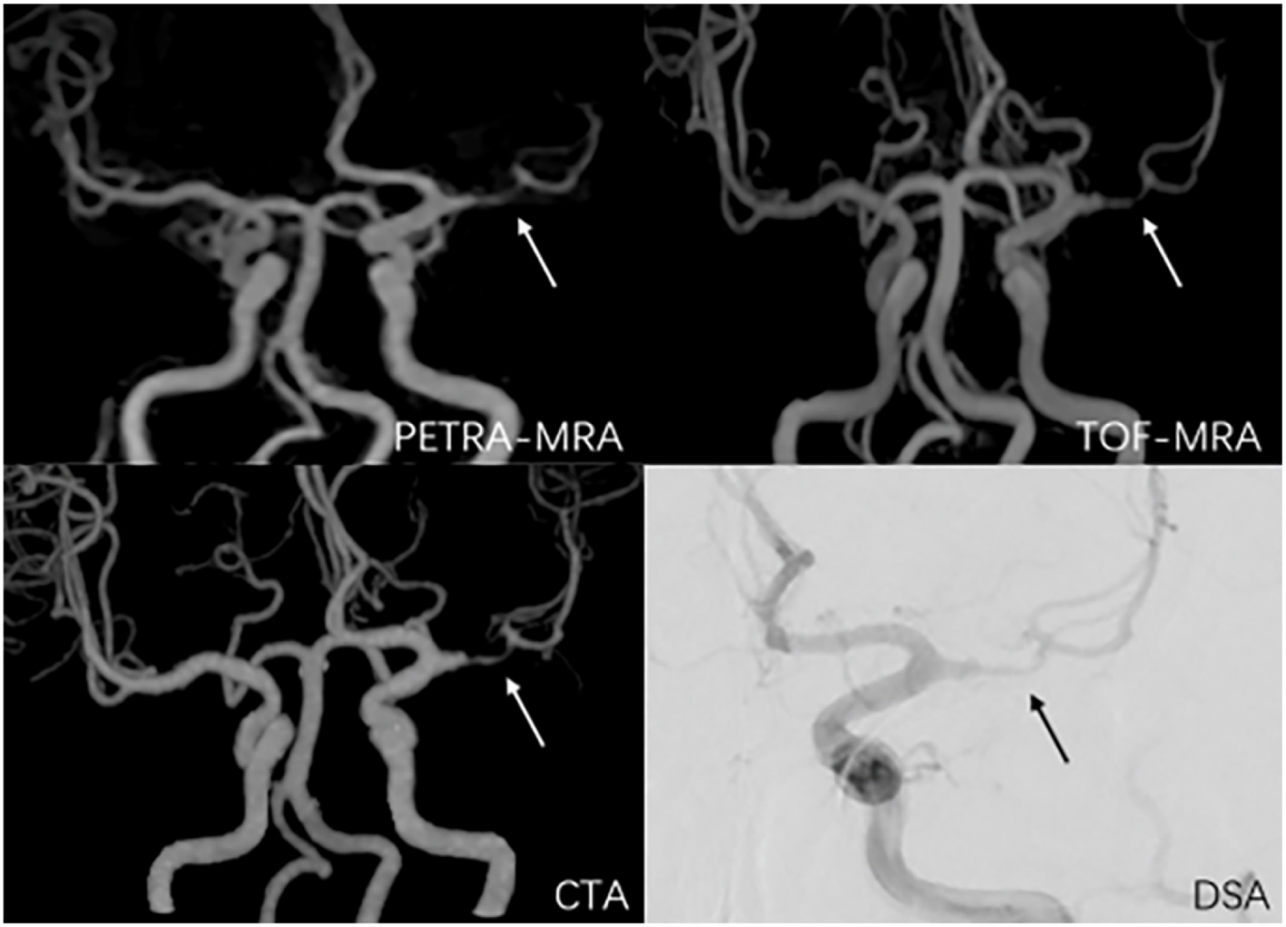
A 38-year-old man with symptomatic left middle artery stenosis. The arrow marks the location of the lesion. The degree of stenosis was 80.9% on PETRA-MRA, 89.1% on TOF-MRA, 88.3% on CTA, and 82.3% on DSA. Lesion length was 6.98 mm on PETRA-MRA, 7.41 mm on TOF-MRA, 7.83 mm on CTA, and 6.5 4 mm on DSA.

**Figure 5:**
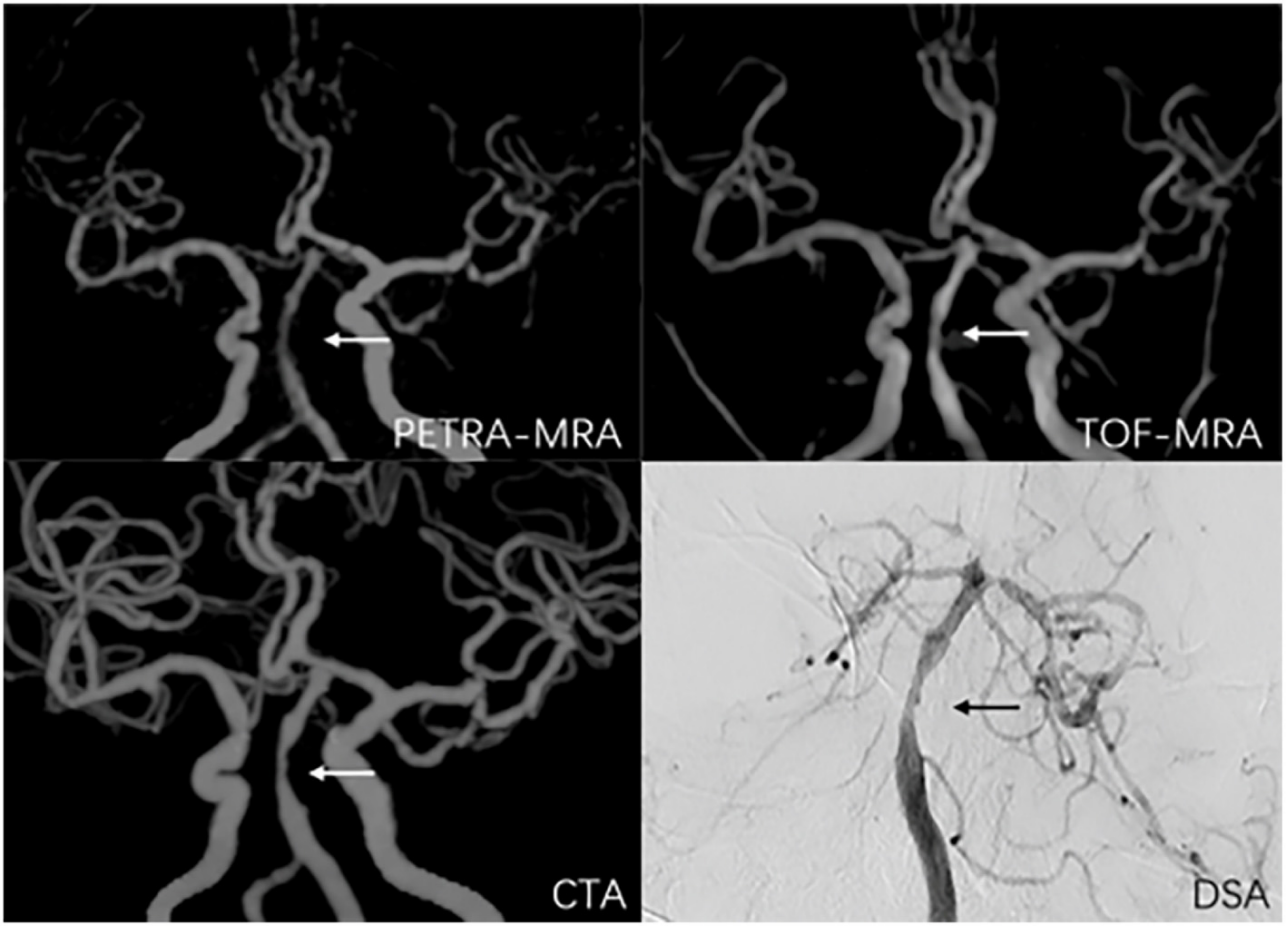
A 56-year-old man with symptomatic basilar artery stenosis. The arrow marks the location of the lesion. The degree of stenosis was 59.9% on PETRA-MRA, 51.1% on TOF-MRA, 69.7% on CTA, and 50.3% on DSA. Lesion length was 5.36 mm on PETRA-MRA, 5.43 mm on TOF-MRA, 5.76 mm on CTA, and 5.05 mm on DSA.

The degree of stenosis acquired with TOF-MRA and CTA was greater than that with DSA (63.0% ± 15.8% vs. 54.0% ± 18.6%, *P* < 0.01; 61.0% ± 18.6% vs. 54.0% ± 18.6%, *P* < 0.01, respectively). However, no marked difference in the degree of stenosis quantification was observed between PETRA-MRA and DSA (55.3% ± 17.7% vs. 54.0% ± 18.6%, *P* = 0.13). No obvious difference in lesion length was noted between PETRA-MRA and DSA (*P* = 0.06), between TOF-MRA and DSA (*P* = 0.17), and between CTA and DSA (*P* = 0.78). The examples from two patients were shown in Figures 4, 5.

With DSA as the reference standard, the sensitivity, specificity, PPV, and NPV of PETRA-MRA, TOF-MRA, and CTA in detecting stenosis of >50% and stenosis of >75% are presented in Table 2. PETRA-MRA achieved the highest value than TOF-MRA and CTA in specificity (94.7% vs. 44.7 and 63.2%) and PPV (96.2% vs. 70.8 and 78.1%) and achieved comparable values of sensitivity (98.1% vs. 98.1 and 96.2%) and NPV (97.2% vs. 94.4 and 92.3%) in detecting stenosis of >50%. Similarly, PETRA-MRA achieved the highest value than TOF-MRA and CTA in specificity (98.7% vs. 78.9 and 85.5%) and PPV (92.3% vs. 38.5 and 56.0%) and achieved the comparable values of sensitivity (85.7% vs. 71.4 and 100.0%) and NPV (97.4% vs. 93.8 and 100.0%) in detecting stenosis of >75%.

**Table 2:** Evaluation of the performance of MRAs and CTA in detecting stenosis of >50% and stenosis of >75% using DSA as the reference standard.

	**PETRA-MRA (+)**	**PETRA-MRA (–)**	**TOF-MRA (+)**	**TOF-MRA (–)**	**CTA (+)**	**CTA (–)**
**Detection of** >**50% stenosis**						
DSA (+)	51	1	51	1	50	2
DSA (–)	2	36	21	17	14	24
Sensitivity (%)	98.1 (89.7–100.0)^a^		98.1 (89.7–100.0)^a^		96.2 (86.8–99.5)^a^	
Specificity (%)	94.7 (82.3–99.4)^a^		44.7 (2861.6–0.7)^a^		63.2 (46.0–78.2)^a^	
PPV (%)	96.2 (87.0–99.5)^a^		70.8 (58.9–81.0)^a^		78.1 (66.0–87.5)^a^	
NPV (%)	97.2 (85.8–99.9)^a^		94.4 (72.7–99.9)^a^		92.3 (74.9.7–99.1)^a^	
**Detection of** >**75% stenosis**						
DSA (+)	12	2	10	4	14	0
DSA (–)	1	75	16	60	11	65
Sensitivity (%)	85.7 (57.2–98.2)^a^		71.4 (41.9–91.6)^a^		100.0 (76.8–100.0)^a^	
Specificity (%)	98.7 (92.9–100.0)^a^		78.9 (68.1–87.5)^a^		85.5 (75.6–92.5)^a^	
PPV (%)	92.3 (64.0–99.8)^a^		38.5 (20.2–59.4)^a^		56.0 (34.9–75.6)^a^	
NPV (%)	97.4 (90.9–99.7)^a^		93.8 (84.8–98.3)^a^		100.0 (94.5–100.0)^a^	

a 95% confidence interval. CTA, computed tomography angiography; DSA, digital subtraction angiography; MRA, magnetic resonance angiography; NPV, negative predictive value; PETRA, pointwise encoding time reduction; PPV, positive predictive value; TOF, time of flight.

**Figure 6:**
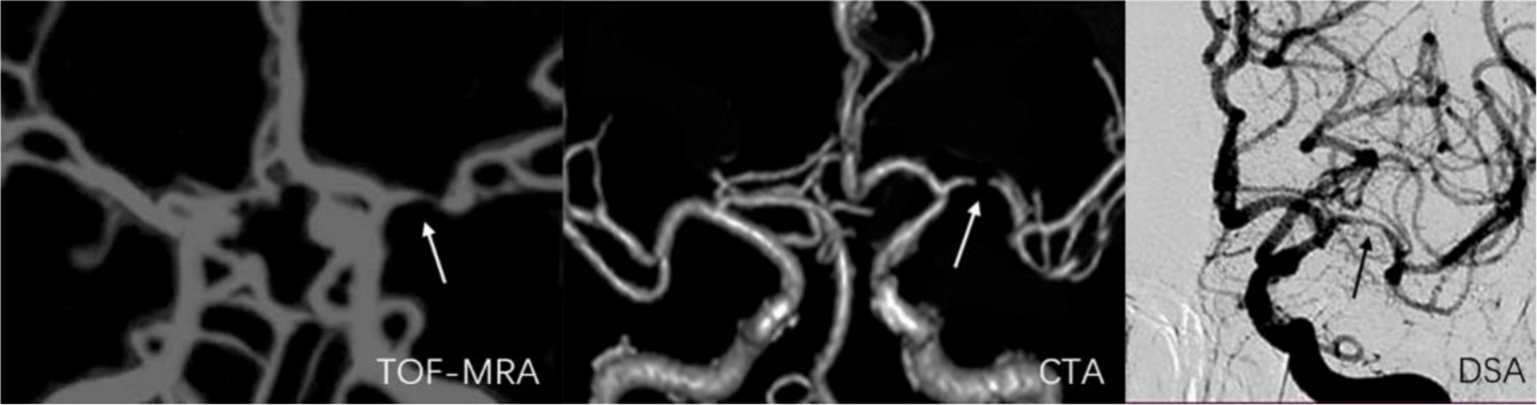
A 55-year-old man with stenosis on the bifurcation and bend of the left middle artery. The arrow marks the location of the lesion. The degree of stenosis acquired on TOF-MRA (75.3%) and CTA (92.5%) exceeded that on DSA (56.9%).

**Figure 7:**
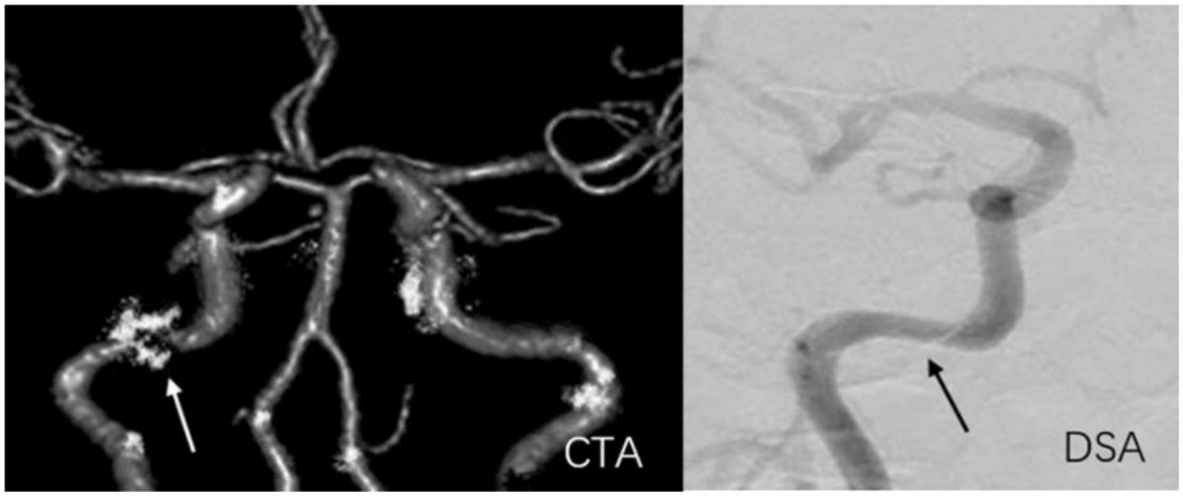
A 59-year-old man with stenosis and calcification on C2 of the right internal carotid artery. The arrow marks the location of the lesion. The degree of stenosis acquired on CTA (81.7%) exceeded that on DSA (50.3%).

There were 52 stenoses (>50%) and 14 stenoses (>75%) according to DSA measurement. The false-positive cases of PETRA-MRA were less than TOF-MRA and CTA in detecting stenosis of >50% (*n* = 2 vs. 21 and 14) and stenosis of >75% (*n* = 1 vs. 16 and 11), and the false-negative cases of PETRA-MRA, TOF-MRA, and CTA were all few in detecting stenosis of >50% (*n* = 1, 1, and 2) and stenosis of >75% (*n* = 2, 4, and 0). There were 21 false-positive cases for TOF-MRA in detecting stenosis of >50% (ACA: MCA: ICA: BA: VA: PCA = 1:12:2:2:1:3), and 70% of stenoses were located at the bend or bifurcation of ACA, MCA, and ICA, such as the junction of M1 and M2 and the internal carotid siphon (Figure 6). There were 14 false-positive cases for CTA in detecting stenosis of >50% (MCA: ICA: BA: VA: PCA= 9: 1: 1: 1: 2), and 30% of stenoses were associated with calcification (Figure 7). The false-negative cases are one stenosis for TOF-MRA in PCA, one stenosis for PETRA-MRA in the intracranial segment of ICA, and two stenoses for CTA (one in MCA and one in PCA). The false-positive cases of PETRA-MRA and the false-negative cases of PETRA-MRA, TOF-MRA, and CTA were relatively small.

With DSA as the reference standard, the performances of PETRA-MRA, TOF-MRA, and CTA on the measurement of the degree of stenosis in severe stenosis, smaller artery stenosis, and anterior/posterior cerebral circulation stenosis are summarized in Table 3. In severe stenosis (>70%), PETRA-MRA was more consistent with DSA than with TOF-MRA and CTA in measuring the degree of stenosis (ICC = 0.78 vs. 0.30 and 0.57), and the degree of stenosis acquired with CTA was greater than that with DSA [82.24% (77.44%, 88.30%) vs. 76.11% (73.60%, 81.75%), *P* < 0.01]. In smaller artery stenosis (M2/A2), PETRA-MRA was more consistent with DSA than with TOF-MRA and CTA in measuring the degree of stenosis (ICC = 0.95 vs. 0.70 and 0.80), and the degree of stenosis acquired with TOF-MRA and CTA was greater than that with DSA (60.94% ± 13.40% vs. 48.20% ± 16.06%, *P* < 0.01; 55.49% ± 18.21% vs. 48.20% ± 16.06%, *P* = 0.03, respectively). In anterior cerebral circulation, PETRA-MRA had a little bigger ICC than TOF-MRA and CTA in measuring the degree of stenosis (0.93 vs. 0.78 and 0.88), and the degree of stenosis acquired with TOF-MRA and CTA was greater than that with DSA [65.79% (54.04%, 77.72%) vs. 54.17 (41.52%, 73.36%), *P* < 0.01; 63.04% (49.93%, 79.17%) vs. 54.17 (41.52%, 73.36%), *P* < 0.01, respectively]. In posterior cerebral circulation, PETRA-MRA had a bigger ICC than TOF-MRA (0.94 vs. 0.71) and the comparable ICC to CTA (0.94 vs. 0.91) in measuring the degree of stenosis, and the degree of stenosis acquired with PETRA-MRA, TOF-MRA, and CTA was greater than that with DSA (48.57% ± 16.29% vs. 45.30% ± 15.78%, *P* = 0.02; 54.97% ± 15.22% vs. 45.30% ± 15.78%, *P* < 0.01; 52.01% ± 18.51% vs. 45.30% ± 15.78%, *P* < 0.01, respectively).

**Table 3:** Comparison of MRAs/CTA and DSA in severe stenosis, smaller artery stenosis, and anterior/posterior cerebral circulation stenosis.

	**DSA**	**PETRA-MRA**	**TOF-MRA**	**CTA**
**Severe stenosis**				
Median (%)	76.11 (73.60, 81.75)^&^	77.05 (70.13, 81.75)^&^	78.41 (74.74, 85.38)^&^	82.24 (77.44, 88.30)^&^
*P*	Reference	0.08	0.56	< 0.01
*ICC*	Reference	0.78	0.30	0.57
**Smaller artery stenosis**				
Mean ± SD (%)	48.20 ± 16.06	50.87 ± 17.56	60.94 ±13.40	55.49 ± 18.21
*P*	Reference	0.07	< 0.01	0.03
*ICC*	Reference	0.95	0.70	0.80
**Anterior cerebral circulation**				
Median (%)	54.17 (41.52, 73.36)^&^	58.37 (42.08, 72.17)^&^	65.79 (54.04, 77.72)^&^	63.04 (49.93, 79.17)^&^
*P*	Reference	0.60	< 0.01	< 0.01
*ICC*	Reference	0.93	0.78	0.88
**Posterior cerebral circulation**				
Mean ± SD (%)	45.30 ± 15.78	48.57 ± 16.29	54.97 ± 15.22	52.01 ± 18.51
*P*	Reference	0.02	< 0.01	< 0.01
*ICC*	Reference	0.94	0.71	0.91

& Interquartile range. CTA, computed tomography angiography; DSA, digital subtraction angiography; MRA, magnetic resonance angiography; PETRA, pointwise encoding time reduction; TOF, time of flight.

### Inter-reader agreement

**Figure 8:**
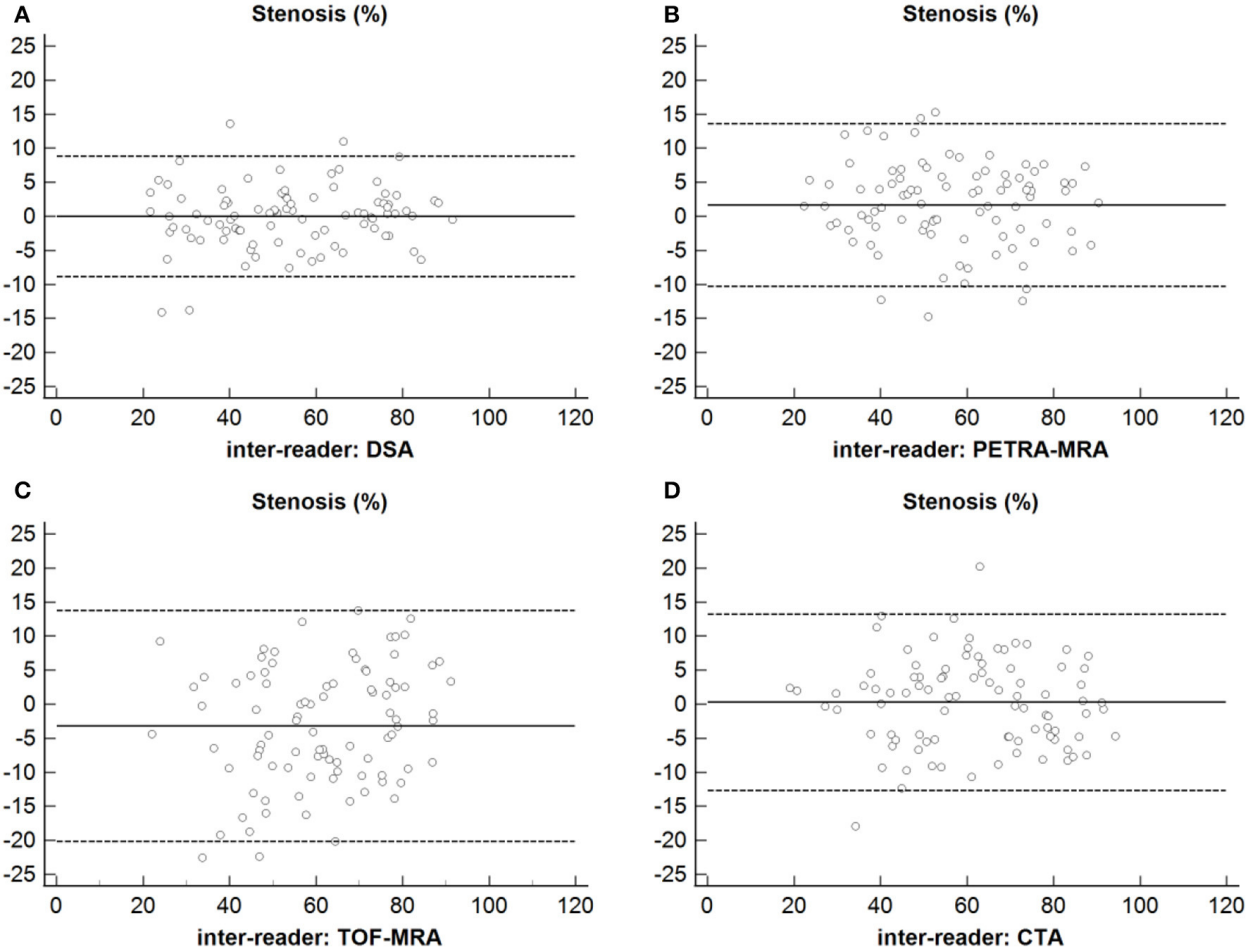
Inter-reader agreement of the measurements of the degree of stenosis using DSA, PETRA-MRA, TOF-MRA, and CTA. **(A)** Stenosis: Inter-reader agreement of DSA. **(B)** Stenosis: Inter-reader agreement of PETRA-MRA. **(C)** Stenosis: Inter-reader agreement of TOF-MRA. **(D)** Stenosis: Inter-reader agreement of CTA.

The excellent inter-reader agreement was observed between two readers for the degree of stenosis and lesion length on PETRA-MRA, TOF-MRA, CTA, and DSA (ICCs >0.80) (Table 4 and Figure 8).

**Table 4:** Comparison of MRAs/CTA and DSA in measuring the degree of stenosis and lesion length.

	**DSA**	**PETRA-MRA**	**TOF-MRA**	**CTA**
**Stenosis (100%)**				
Reader 1	54.0 ± 18.6	55.3 ± 17.7	63.0 ± 15.8	61.0 ± 18.6
Reader 2	54.0 ± 19.1	57.0 ± 17.2	59.8 ± 17.5	61.3 ± 18.3
CV (%)	8.4	10.9	14.1	10.8
Bias	−0.02	1.7	−3.2	0.3
LOA	(−8.9, 8.8)	(−10.3, 13.6)	(−20.2, 13.7)	(−12.6, 13.2)
*ICC*	0.97	0.94	0.86	0.94
**Lesion length (mm)**				
Reader 1	3.12 (1.84, 4.30)	3.21 (2.04, 4.27)	3.30 (2.07, 4.20)	2.68 (1.52, 4.64)
Reader 2	3.12 (1.73, 4.18)	3.23 (1.89, 4.21)	3.30 (1.97, 4.40)	2.60 (1.51, 4.66)
*ICC*	0.99	0.99	0.99	0.97

CTA, computed tomography angiography; CV, coefficient of variation; DSA, digital subtraction angiography; ICC, intraclass coefficient; LOA, limit of the agreement; MRA, magnetic resonance angiography; PETRA, pointwise encoding time reduction; TOF, time of flight.

## Discussion

Using DSA as the reference standard to compare PETRA-MRA, TOF-MRA, and CTA in the degree of stenosis and lesion length, we demonstrated that TOF-MRA and CTA both overestimated the degree of stenosis compared with DSA. PETRA-MRA was in closest agreement with DSA and over-performed TOF-MRA and CTA, and the results were still the same in severe stenosis, smaller artery stenosis, and anterior/posterior cerebral circulation stenosis. PETRA-MRA achieved the highest specificity and PPV in detecting stenosis of >50% and stenosis of >75%. Moreover, the imaging quality and inter-reader reproducibility were excellent for PETRA-MRA. Therefore, PETRA-MRA may be a robust non-invasive imaging option for the precise measurement of luminal stenosis. To the best of our knowledge, this is the first prospective study that directly compares PETRA-MRA with CTA and 3D TOF-MRA using DSA as a gold standard. The prospective study design allows us to perform four imaging methods in the same cohort of patients, and such direct comparison was valuable to understand the advantages and drawbacks of each imaging method and help to choose the best imaging method for each patient.

Computed tomography angiography is a very popular technique for intracranial stenosis evaluation because it has a fast scan time and good image quality. This study found that CTA overestimated the degree of stenosis compared with DSA even in severe stenosis, smaller artery stenosis, and anterior/posterior cerebral circulation stenosis. This may attribute to that CTA is sensitive to intracranial atherosclerotic plaque calcification and eddy current effect in the stenosis area, which increases the volume effect of local imaging, resulting in the overestimation of stenosis (17–19). Intracranial atherosclerotic plaque calcification occurs mainly in the internal carotid artery and vertebral-basilar artery. Several false-positive cases that occurred in ICA and VA had stenoses with calcification in the study. Mannil et al. reported that compared with conventional CTA, a modified dual-energy CT algorithm achieved higher consistency with DSA by eliminating blooming artifacts due to calcification (20). Moreover, this study also found that CTA achieved a smaller specificity in detecting stenosis of >50% than stenosis of >75%. This is consistent with previous studies that the specificity of CTA in assessing stenosis remains low in patients with moderate stenosis (21). In addition, CTA requires the introduction of exogenous contrast agents, which should be used with caution in patients with renal insufficiency. Therefore, these limitations of CTA should be considered carefully in routine clinical use (4).

In this study, the degree of stenosis obtained with TOF-MRA exceeded the measurement using DSA. The same result also occurred in the smaller stenosis group, anterior cerebral circulation, and posterior cerebral circulation stenosis group. This phenomenon could be explained by which TOF-MRA was sensitive to the flow velocity and direction of blood flow, especially in the origin of arteries, vessel bifurcations, curved vessels, and vessels parallel to the cross-sectional plane (22–24). This study found the false-positive cases of TOF-MRA mainly were located at the bifurcations and bend of the cerebral artery. Tian et al. reported a similar finding that TOF-MRA overestimated the degree of stenosis relative to DSA (25). Sarikaya et al. also demonstrated that the overestimation of the degree of stenosis by TOF-MRA was more common than that by intracranial vessel wall MRI (26).

The measurements of the degree of stenosis and lesion length acquired on PETRA-MRA were in closest agreement with those on DSA compared with TOF-MRA and CTA, and PETRA-MRA achieved the highest specificity and PPV in detecting stenosis. For severe stenosis, the smaller artery stenosis, anterior/posterior cerebral circulation stenosis, and the degree of stenosis acquired on PETRA-MRA were also in closest agreement with those on DSA compared with TOF-MRA and CTA. These are closely related to the principle of PETRA-MRA. The PETRA sequence combined radial and Cartesian acquisitions of the k-space, rendering PETRA-MRA less prone to flow artifacts and achieving high signal homogeneity and increased SNR (9, 27). The ultrashort echo time (TE <100 μs) applied to PETRA-MRA was insensitive to phase dispersion because the phase errors generated during longer TE were larger (28, 29). Furthermore, PETRA-MRA was unsusceptible to the direction of blood flow due to employing an endogenous contrast tracer to label the inflowing blood magnetically (30). These technical advantages of PETRA made its imaging quality better, which can more accurately assess lesions, especially the bifurcation and bend of the cerebral artery and the distal small artery. Shang et al. revealed that zero TE-MRA (a technique comparable to PETRA-MRA) had excellent image quality and performance in depicting cerebrovascular diseases (8). Zhang et al. also reported that PETRA-MRA had higher image quality and agreement with DSA in measuring the degree of stenosis of the middle cerebral artery than TOF-MRA (13). Our study agrees well with these previous studies. For posterior cerebral circulation stenosis, PETRA-MRA had significantly different from DSA in measuring the degree of stenosis, which may be related to the less sample.

The main disadvantage of PETRA-MRA is the longer scan time (9 min and 20 s) relative to TOF-MRA (3 min and 29 s). The long scan time of PETRA-MRA would increase the risk of motion artifacts, reduce image quality, and limit its application in clinical practice even with the high accuracy for ICAS assessment. However, in this study, only five of 72 included patients (7%) had poor image quality due to motion, which was acceptable. The reason was that the radial acquisition was inherently not sensitive to motion because of the oversampling of the center of k-space compared with Cartesian sampling (31). In addition, the acoustic noise levels of PETRA-MRA were obviously lower than those of TOF-MRA (~59 vs. 73 dB). The low acoustic noise could eliminate patient anxiety and increase patient compliance. This can help to reduce motion artifacts and increase the success rate of the scan. Especially, this feature is very friendly for pregnant women, infants, and mental patients who are sensitive to sound. In addition, there have been under-sampling and sparse reconstruction techniques for radial MRI that can significantly reduce the scan time by more than 50% (31, 32), but unfortunately, these techniques were only available in a few research centers because most vendors have not commercialized these techniques and most of the advance technique requires GPU hardware (not available in most clinical scanners). With advances in hardware and reconstruction techniques, we believe that accelerated PETRA-MRA in <5 min will be available soon and overcome the current barrier of long scan time.

This study had certain limitations. First, this study covered only single-center data with small sample size. Larger-scale multi-center studies are needed to confirm our findings. Second, only 2D DSA was used in this study with limited view angles. 3D rotational DSA should be used in future studies to act as the true gold standard of stenosis measurement. Finally, this technology of PETRA-MRA should be improved constantly to deal with the shortcoming of long scan time.

## Conclusion

PETRA-MRA is more accurate than TOF-MRA and CTA for the evaluation of intracranial stenosis and lesion length when using DSA as a reference standard. Particularly, PETRA-MRA also has certain advantages in the evaluation of smaller artery stenosis and severe stenosis. In summary, PETRA-MRA is a promising non-invasive tool for ICAS assessment.

## Data availability statement

The raw data supporting the conclusions of this article will be made available by the authors, without undue reservation.

## Ethics statement

Written informed consent was obtained from the individual(s) for the publication of any potentially identifiable images or data included in this article.

## Author contributions

JN, YR, RC, FZ, and XW helped in recruiting patients. JN, YR, YaZ, and CZ conceived and designed the study and performed data analysis and interpretation. All authors made a substantial contribution to data research, discussion of content, reviewing and editing of the manuscript before submission, read, and approved the final manuscript.
